# Supplementary material for: Association between oral microbiome and seven types of cancers in East Asian population: a two-sample Mendelian randomization analysis
Source: Front Mol Biosci. 2023 Nov 21;10:1327893. doi: 10.3389/fmolb.2023.1327893 (PMC10702768; doi:10.3389/fmolb.2023.1327893)
Supplement: Supplementary file 1 [file DataSheet1.ZIP › Supplementary Materials/MR plots of saliva/saliva═╝/colorectal cancer/pheno.2883_to_colorectal cancer_leave_one_out.pdf]

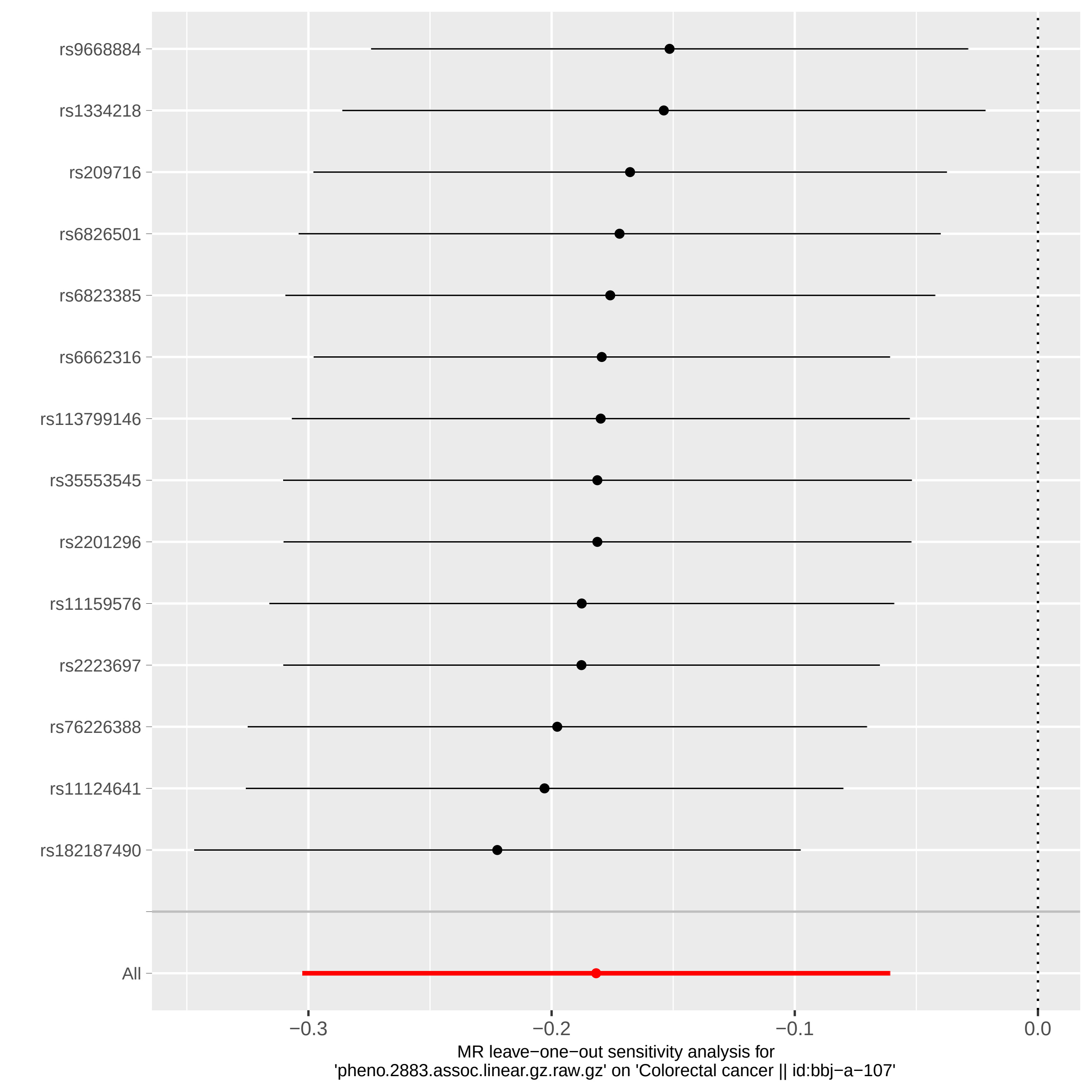
rs9668884
rs1334218
rs209716
rs6826501
rs6823385
rs6662316
rs113799146
rs35553545
rs2201296
rs11159576
rs2223697
rs76226388
rs11124641
rs182187490
All
−0.3
−0.2
−0.1
0.0
MR leave−one−out sensitivity analysis for
'pheno.2883.assoc.linear.gz.raw.gz' on 'Colorectal cancer || id:bbj−a−107'
